# RNA Viral Vectors for Accelerating Plant Synthetic Biology

**DOI:** 10.3389/fpls.2021.668580

**Published:** 2021-06-23

**Authors:** Arjun Khakhar, Daniel F. Voytas

**Affiliations:** ^1^Department of Genetics, Cell Biology and Development, University of Minnesota, St. Paul, MN, United States; ^2^Center for Precision Plant Genomics, University of Minnesota, St. Paul, MN, United States; ^3^Center for Genome Engineering, University of Minnesota, St. Paul, MN, United States

**Keywords:** RNA viruses, RNA viral vectors, gene editing, synthetic biology, plant synthetic biology, synthetic transcription factors, viral engineering

## Abstract

The tools of synthetic biology have enormous potential to help us uncover the fundamental mechanisms controlling development and metabolism in plants. However, their effective utilization typically requires transgenesis, which is plagued by long timescales and high costs. In this review we explore how transgenesis can be minimized by delivering foreign genetic material to plants with systemically mobile and persistent vectors based on RNA viruses. We examine the progress that has been made thus far and highlight the hurdles that need to be overcome and some potential strategies to do so. We conclude with a discussion of biocontainment mechanisms to ensure these vectors can be used safely as well as how these vectors might expand the accessibility of plant synthetic biology techniques. RNA vectors stand poised to revolutionize plant synthetic biology by making genetic manipulation of plants cheaper and easier to deploy, as well as by accelerating experimental timescales from years to weeks.

## Introduction

Synthetic biology promises to be transformative to biological science^1^ by providing two major new approaches to interrogate biological mechanisms. Firstly, by allowing molecular interventions of previously impossible precision and tunability using synthetic signaling systems (Havens et al., 2012; Khakhar et al., 2016; Nemhauser and Torii, 2016) and/or genome engineering (Rodríguez-Leal et al., 2017; Zsögön et al., 2018), it has allowed us to make controlled perturbations to natural systems to build, test, and validate mechanistic models of biology. Secondly, by engineering previously characterized biological components into novel configurations with predicted behaviors, such as the creation of genetic circuits (Sprinzak and Elowitz, 2005; Antunes et al., 2006, 2011; Khakhar et al., 2018), we can test and refine our understanding of these components and the broader cellular and organismal context they operate in. These two approaches have led to some major breakthroughs in understanding microbial (Sprinzak and Elowitz, 2005) and mammalian biology (Mathur et al., 2017) and can be applied to diverse biological systems, including plants (Patron, 2020).

Synthetic biology approaches have been used to elucidate mechanistic rules behind core developmental and stress response pathways, such as auxin regulated development (Pierre-Jerome et al., 2014; Moss et al., 2015) and abscisic acid triggered drought responses (Park et al., 2015). Another major area of focus has been uncovering the rules governing promoter architecture utilizing synthetic biology both to study a diversity of elements in a massively parallel fashion (Cai et al., 2020), and to study how targeted variation in promoter architectures affect gene transcription (Antunes et al., 2009; Belcher et al., 2020) and associated phenotypes. Some of these insights coupled with innovations in modular plasmid assembly and synthetic transcription factors have enabled a new era of metabolic engineering with applications as diverse as enhancing photosynthesis (South et al., 2019), producing valuable small molecules (Moses et al., 2013), and providing auto luminescence (Khakhar et al., 2020).

**FIGURE 1:**
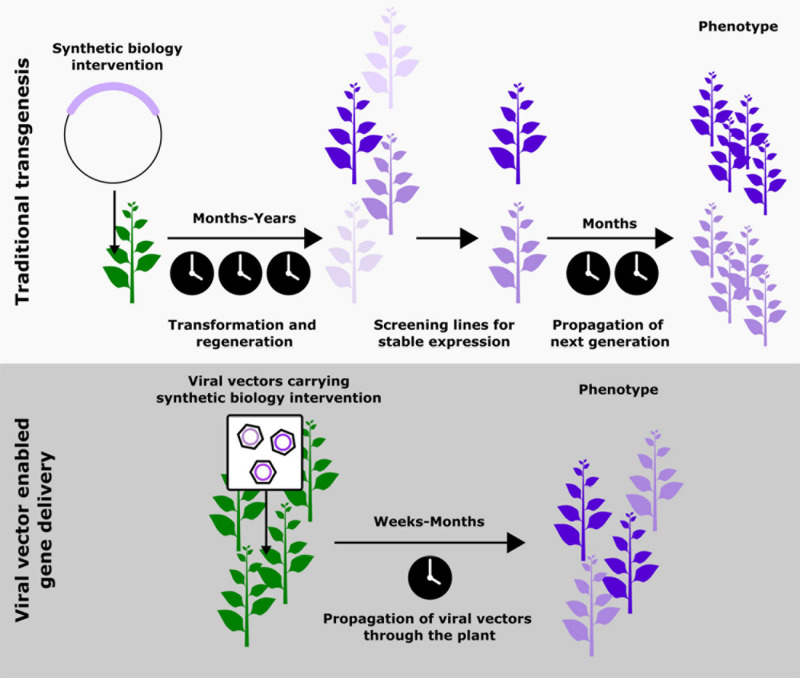
Schematic comparing the steps and approximate timescales associated with traditional transgenesis and viral vector mediated gene delivery as a means to implement synthetic biology-based modifications in plants. The top panel is adapted from Khakhar et al. (2021). Copyright American Society of Plant Biologists; www.plantphysiol.org.

When the advances in plant synthetic biology are studied closely, it becomes apparent that the bulk of risky exploratory work is done using transient expression systems; stable transgenesis is reserved for more well studied pathways. This trend is likely because generating stable transgenic lines, especially outside model species like *Arabidopsis thaliana*, can take years, be prohibitively costly, and require specialized technical expertise (Altpeter et al., 2016) (Figure 1). Recent innovations involving the use of developmental regulators to aid transformation are making significant improvements to this process (Gordon-Kamm et al., 2019), most significantly expanding the plants that can be transformed from a small set of lines in model species. However, this technology does not overcome one of the biggest contributors to the timescale of transformation, the generation time of the plant, which can vary from several months to several years. Hence, the most popular solution to this issue currently is the transient expression of synthetic biology interventions (Antunes et al., 2009; Shih et al., 2016). While these strategies have enabled more rapid prototyping, they are limited by the inability to allow the study of biological phenomenon that occur over periods of time longer than a few days or across different tissues in a plant. An ideal solution would be a cheap and easy-to-use tool that allows the delivery and maintenance of foreign genetic material systemically in an adult plant. To realize this ideal outcome, it is first necessary to reject a dogma that has become pervasive: that persistent expression necessitates genomic integration of the transgene. Inspiration to design this ideal tool can be taken from one of nature’s most proficient plant engineers, plant viruses.

Using plant viruses to deliver synthetic biology interventions could overcome the time scale and cost associated with transgenesis, as well as the inability to study whole plant phenotypes associated with transient expression (Figure 1). By moving away from genomic integration of transgenes drawbacks associated with transgenesis, such as chromatin context, generational silencing and insertional variation of transgenes can also be avoided. However, these benefits do come with some challenges, namely potentially confounding host responses to the virus, limited cargo capacity, and viral silencing by the plant. We will examine these in greater detail later in in the review and suggest some potential solutions.

## Plant Viruses for Engineering Plants

Plant viruses have the capacity to deliver foreign genetic material systemically throughout the plant. Expression of this genetic material can be maintained over long periods of time, and, in some cases, the plants are completely asymptomatic (Macfarlane, 2010; Pasin et al., 2019). There are a plethora of DNA and RNA viruses that have been explored as gene delivery tools, each with their own strengths and weaknesses. Geminiviruses, which are single stranded DNA viruses, normally have limited cargo capacities. However, their cargo capacity can be augmented using geminivirus-derived replicons, which enables delivery of relatively large DNA cargos at high concentrations. This capacity has been leveraged to deliver high-copy repair templates for genome editing purposes (Čermák et al., 2015). However, this large cargo capacity comes at the cost of mobility. These viruses have also been reported to perturb the plants cell cycle as part of their replication process (Ruhel and Chakraborty, 2019), which might make them a sub-optimal tool to deliver reagents to study native biological phenomenon. Single stranded RNA viruses do not suffer from this drawback. These viruses have either positive or negative stranded genomes packaged within their capsids. Of these, positive strand RNA viruses have been more extensively studied, making engineering them for gene delivery straightforward (Macfarlane, 2010; Pasin et al., 2019). Additionally, they require no pre-existing proteins to initially facilitate infection, unlike negative single strand viruses (Ma et al., 2020).

Using viral vectors to deliver foreign genetic cargo is not a new idea, it has been the predominant strategy for gene delivery in mammalian systems for decades (Zhang and Godbey, 2006; Lentz et al., 2012). This strategy has been applied to plants as well, as recently reviewed by Pasin et al. (Pasin et al., 2019). In general, developing viral vectors involves identifying a region of the viral genome that is amenable to the insertion of an additional coding sequence, which is either expressed in tandem with a viral protein and post-translationally separated with a 2A peptide or expressed from a sub-genomic promoter. The bulk of virus aided interrogation of plant biology has focused on the delivery of reagents to silence endogenous gene expression rather than gene delivery. In this approach, called viral induced gene silencing (VIGS; Liu et al., 2002; Burch-Smith et al., 2006), the natural capacity of plants to silence viral sequences is redirected to target endogenous mRNA by encoding part of its sequence in the viral genome. VIGS has aided the elucidation of biological phenomenon by enabling rapid, transgenesis free, functional genetics in a broad range of species from legumes to fruit trees (Velásquez et al., 2009; Sasaki et al., 2011; Lange et al., 2013; Pflieger et al., 2013). The current state of the art was recently reviewed by Dommes et al. (2018).

The use of plant viruses for gene delivery has thus far been primarily focused on the production of biologics in plants (Gleba et al., 2005, 2007; Dugdale et al., 2014). In all these approaches the prodigious replication of RNA viruses is leveraged to maintain a gene coding sequence inserted into the viral genome at high concentration and thus enable strong protein expression. While such over expression techniques have been widely applied in industry (Giritch et al., 2017), their application to the interrogation of biological phenomena has been limited. There have been a few major examples of how this approach can be used to deliver genes to elucidate aspects of fruit ripening (Zhou et al., 2012), anthocyanin biosynthesis (Bedoya et al., 2012), and carotenoid biosynthesis (Llorente et al., 2020). For example, Llorente et al. (2020) were able to establish that loss of photosynthetic competence and enhanced carotenoid production were required for chromoplast development via overexpression of the crtB protein from a plant RNA viral vector. Recent work by Torti et al. (2021) demonstrated that single genes could be delivered to a range of plants via RNA viral vectors to create several different agronomically important traits including dwarfing and flowering time. They used a combination of whole plant delivery techniques via high pressure spraying, modified versions of potato virus X that included additional silencing machinery, and modified cargos with higher GC content to achieve these results.

The relative rarity of these successful applications is likely due to the loss of mobility of these vectors when loaded with large cargos, limiting their delivery capacity (Pasin et al., 2019). This has precluded their use for the delivery of sufficiently large cargos for some common synthetic biology interventions, which use large proteins such as synthetic transcription factors. Additionally, these viruses tend to move in a non-uniform, “patchy” manner (Bedoya et al., 2012), which is suboptimal if the phenomenon being studied requires uniform gene delivery. However, recent work that has married synthetic virology and plant synthetic biology has led to some exciting innovations that overcome some of these challenges to accelerate the study of plant biology.

## Accelerating Plant Synthetic Biology With RNA Viral Vectors

One successful strategy to overcome cargo capacity limitations of RNA viral vectors has been to leverage the two-component nature of CRISPR-Cas systems. These systems consist of a large protein component that can act as a nuclease or be engineered to behave as a DNA binding domain, as well as a small RNA molecule called a guide RNA (gRNA). This gRNA associates with the protein to guide it to specific region of the genome. While the protein component is too large to load onto most viral vectors, the gRNA is not. Thus, it is possible to create transgenic plant lines that constitutively express large RNA guided enzymes and deliver the gRNAs that direct these enzymes with viral vectors.

This strategy has been used by several groups to achieve high efficiency somatic genome editing (Cody and Scholthof, 2019; Ellison et al., 2020; Ma et al., 2020; Uranga et al., 2021). Plants lines that constitutively express the programmable nuclease, Cas9, were treated with vectors that systemically deliver gRNAs, which direct Cas9 to cut a specific genomic locus. Initial attempts were restricted to the editing to somatic cells, however, through engineering improved viral movement efficiency, heritable editing has been achieved (Ellison et al., 2020; Uranga et al., 2021). The incorporation of movement enhancing tRNA-like sequences (Kehr and Kragler, 2018) dramatically increased somatic editing efficiencies and enabled editing to occur in germ line cells. Mutations were propagated to the next generation with efficiencies of greater than 90% in the model plant *Nicotiana benthamiana*.

In addition to genome editing, site specific recombination via recombinases is another strategy that holds great promise to enable dissection of the links between genotype and phenotype. These recombinase enzymes, such as the commonly used Cre protein, are capable of recognizing a specific sequence of DNA and catalyzing a directional DNA exchange reaction. It has been demonstrated that recombinases can be delivered via RNA viruses, and that the predictable recombination events can be inherited by the progeny of the infected plants (Kopertekh et al., 2004). As viral genome editing is extended to other plants it has the potential to obviate the need for transgenesis to create genome edited plants and thereby dramatically accelerate genome editing-based interrogation of plant biology.

The use of RNA viruses has also been successfully applied to somatic transcriptional reprogramming. Plant lines that stably expressed a Cas9-based transcription factor were created, and viral vectors were used to deliver gRNAs to target the transcription factor. This strategy was used to tune the expression of metabolic and developmental master regulator genes and study associated phenotypes in weeks as compared to the months to years it would take with traditional transgenesis. This strategy was also applied to enable targeted methylation in somatic tissue as well as germline tissue to create heritable phenotypes (Ghoshal et al., 2020). One limitation of all these approaches is that the direct fusion of the Cas9 protein to the transcription or methylation effector domains restricts the kinds of perturbations that the viral vectors can implement. A promising solution to this dilemma is the constitutive expression of a toolbox of effectors which can be assembled into the desired activators or repressors *in planta* via RNA scaffolds (Zalatan et al., 2015) that are delivered on viral vectors. An even more flexible approach that has been demonstrated is to co-deliver both the effectors and gRNA scaffolds via an ensemble of vectors to a plant stably expressing just the Cas9 protein. Here too the inclusion of movement enhancement motifs was found to be essential for effective regulation, likely because it enables high frequency colocalization of vectors within the ensemble. While this approach was able to create measurable changes in plant phenotype, the fold changes in gene expression observed were relatively modest, pointing to a large potential for optimization of this strategy. These approaches have been successfully applied to study both metabolic and developmental pathways in a range of plants, including the model plants *A. thaliana*, *N. benthamiana*, and the crop plant *Solanum lycopersicum*.

**FIGURE 2:**
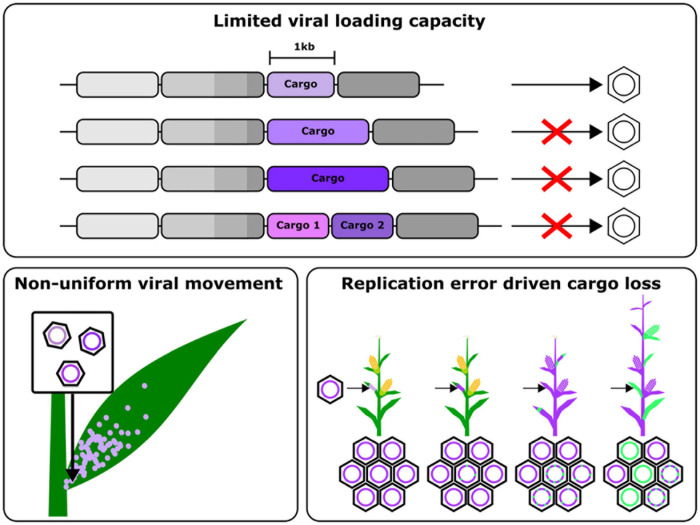
Schematic summarizing the major challenges that need to overcome to engineer improved viral gene delivery tools, namely, a limited cargo capacity, non-uniform viral movement in plants and cargo loss due to error prone replication and recombination.

The above approaches, however, are still limited by the inability to load large cargos onto viral vectors, necessitating genomic integration of proteins like Cas9 (Figure 2). Some groups have leveraged negative single stranded RNA viruses, which have larger cargo capacities, to overcome this issue, however these still suffer from the drawbacks of requiring several accessory proteins to establish an infection mentioned earlier. While the use of movement enhancement motifs has been shown to improve the uniformity of viral cargo delivery, this still remains an area of viral engineering with many open questions. Finally, these viruses are also susceptible to silencing, leading to a gradual decline in the expression of the delivered cargo. These challenges highlight some of the engineering opportunities to improve viral vectors to the point that they may one day replace stable transgenesis as the primary means to deploy the tools of plant synthetic biology. In the rest of this review, we examine the aspects of RNA viral vectors that need to be reengineered and some promising strategies to do so (Figure 2).

## Enhancing Viral Vector Movement

Single stranded RNA viruses move through plants in two major ways: in a local fashion through the cell-to-cell connections, the plasmodesmata (Swanson et al., 2002; Morozov and Solovyev, 2003; Solovyev and Savenkov, 2014), and systemically via the vasculature (Swanson et al., 2002; Morozov and Solovyev, 2003; Solovyev and Savenkov, 2014). These two modes of movement operate on different time scales and are thought to be driven by different mechanisms. Local movement through plasmodesmata is a slow process and is driven by viral movement proteins that play dual roles of shuttling the viral genome to the plasmodesmata as well as increasing the size exclusion limit of this pore to facilitate cell-to-cell movement (Waigmann et al., 1994; Morozov and Solovyev, 2003). Systemic movement occurs when the virus is able enter the vasculature, at which point its movement seems to follow source-sink dynamics as it is rapidly trafficked across the plant. For some viruses this systemic movement is thought to be driven, in part, by the presence of tRNA-like sequences in the viral genome (Zhang et al., 2016). Similar sequences have been identified in mobile plant RNAs such as the transcripts of the *FT* gene, which are signals that travel up the shoot to trigger flowering, and the *BEL5* gene, an RNA that travels to the root and trigger tuber formation in potatoes (Zhang et al., 2016). In addition, tRNAs themselves have been shown to be mobile from cell-to-cell (Zhang et al., 2009). The precise molecular mechanism of how these motifs enable vascular movement is still unclear but is thought it involve interaction with phloem loading proteins (Kehr and Kragler, 2018).

While viruses can move systemically through the plant from the initial point of delivery, this movement becomes increasingly patchy and non-uniform at progressively distal points (Bedoya et al., 2012). The viruses might simply not need to move uniformly to mount a productive infection or the plants capacity to silence the virus may not be uniformly effective across the plant (Devers et al., 2020). Using viruses as effective gene delivery tools will require uniform and rapid spread of the cargo from the point of infection throughout the plant. One potential avenue to achieve this is to identify more efficient variants of the movement proteins, through processes such as directed evolution, or by screening natural variants (Kearney et al., 1999; Borniego et al., 2016). However, as these proteins have several functions and interact with several host proteins, any changes to their function will likely affect several aspects of viral biology, making them non-ideal engineering targets. It has been empirically observed by us and others that incorporating additional exogenous tRNA-like sequences into viral genomes can improve systemic movement efficiency (Ellison et al., 2020; Ghoshal et al., 2020). These sequences have also been demonstrated to enable penetration of previously inaccessible tissues such as the meristem, which is of special relevance for genome engineering applications (Li et al., 2011). The movement enhancement, observed as more uniform systemic delivery, seems to occur across different viruses and in different plant hosts, indicating this may be a host- and vector-independent strategy to engineer viral movement (Zhang et al., 2016). This strategy represents a modular way to achieve enhanced movement with minimal effects on other aspects of viral biology. It still remains to be explored if the enhancement derived from different tRNA-like sequences is synergistic or additive. It is also an open question which tRNA-like sequences confer the strongest movement enhancement, and if the speed of movement through the plant and uniformity of movement through a tissue are related.

An important nuance of engineering viral mobility is the impact of virus exclusion, which is when infection of a cell by a particular plant virus prevents the infection by other viruses (Zhang et al., 2018). Certain experiments may demand delivery of cargos larger than the capacity of a single vector. In these situations, some groups have used complementary viruses that can co-infect cells (Giritch et al., 2006), which is a viable strategy but sometimes leads to extreme viral symptoms (Malapi-Nelson et al., 2009; Gil-Salas et al., 2012). An alternative approach we have demonstrated involves co-delivery of multiple versions of the same viral vector with different cargos (Khakhar et al., 2021). The inclusion of tRNA-like motifs into these vectors conferred a significant improvement of co-localization of multiple cargos in systemic tissues, where segregation of co-delivered cargos normally occurs. However, it is still not clear if these improvements are indicative of co-infection by the viral vectors, or sub-genomic RNAs generated from them that are trafficked from neighboring cells. This highlights the importance of further enhancement of viral movement, as it would enable not just an increase in the uniformity of delivery but also in the overall cargo capacity via the use of ensembles of vectors based on the same virus carrying different cargos.

Another exciting avenue of exploration is the potential for engineering viral movement for tissue specific delivery. It has been observed that certain viruses specifically localize to certain tissues (Rothenstein et al., 2007; Harper et al., 2014). This may, in part, be driven by targeted vascular unloading of viral genomes into particular tissue types. This sort of tissue specific RNA movement can be seen in endogenous mRNAs that contain tRNA-like sequences such as BEL5 and FT, which are specifically transported toward the root and meristem respectively (Kehr and Kragler, 2018). One hypothesis to explain this is that differences in the secondary structure of these sequences lead to specific associations with host proteins that target their selective movement. The design rules that govern the tissue specificity of these motifs are poorly understood. Elucidating them would enable the creation of more sophisticated vectors to precisely deliver genetic cargos and create tissue specific phenotypes. This, in turn, would allow the interrogation of pathways where systemic expression would confound experiments or prove lethal. While a lot remains to be learned about these motifs, they represent a powerful way to modularly engineer viral movement in plants.

## Engineering Viral Vector Replication

The replication of RNA viral vectors is an important determinant of their capacity to enable stable gene expression. RNA viruses are notorious for having relatively large error rates during replication in the order of 10^–5^ to 10^–6^ mutations per site per generation (Tromas and Elena, 2010), which would lead to a little under 10% of the genomes produced in an infected cell being mutants (Martínez et al., 2011). This is thought to be an evolutionary strategy that, when paired with their rapid replication rates, enables them to quickly find avenues around their host’s defense responses (Lauring and Andino, 2010). However, this also leads to a less stable cargo, as accumulating non-synonymous mutations or recombination will eventually lead to cargo loss. One avenue to improve the fidelity of the RNA dependent RNA polymerase (RdRp), a core component of the replicase of all plant RNA viruses, is to make specific mutations to the active site. This approach has been demonstrated to allow significant improvements in fidelity in a study on poliovirus (Shi et al., 2019). The high degree of conservation of RdRp structure and function across viruses implies these or similar mutations, and certainly the broad approach, could be applied to plant viruses for vector improvement. These higher fidelity replicases would allow cargo delivered by these vectors to persist for longer, enabling the study of biological phenomena over longer timescales.

Replication speed is also not necessarily in line with an effective gene delivery platform. Viral replication speed has evolved to optimize the propagation of the virus within the host and between hosts (Elena et al., 2008). However, for optimal gene delivery, we hypothesize that viral replication needs sit in an intermediate sweet spot. It must be fast enough to overcome silencing and ensure efficient replication throughout the plant, while not being so rapid that it causes significant physiological disruptions and associated severe viral symptoms. As the current standard for the development of viral vectors for a new host is to bio-prospect for a virus that naturally occupies this sweet spot (Pasin et al., 2019), a large fraction of vectors in use today have suboptimal aspects related to ease of infection or pathogenic symptoms. This balance is also affected by environmental conditions, potentially due to modulation of both replication rate and silencing by temperature (Zhao et al., 2016). One avenue to engineer around this challenge is to use our current understanding of the processivity of RdRps (Korneeva and Cameron, 2007; Draghici et al., 2009) and apply protein engineering techniques to tune replication speed. Another exciting possibility is to engineer the secondary structure of the motifs recognized by the viral replicase to modulate the association of the replicase with the viral genome (Mueller et al., 1997; Ishibashi and Ishikawa, 2015), and thereby titrate replication speed. The capacity to be able to tune replication speed would not only enable the creation of more effective gene delivery tools with minimal host response, but it would also allow novel applications of synthetic biology. For example, the strength of regulation implemented by a delivered synthetic transcription factor could be altered by tuning replication speed of the vector, to study the relationship between gene expression and developmental phenotypes (Khakhar et al., 2018). This same approach could be used to titrate the levels of a delivered enzyme and study its impact on metabolic flux (Belcher et al., 2020), a crucial aspect to metabolic engineering.

## Engineering Delivered Cargo Sequences

An important aspect in designing vectors for effective gene delivery that is often overlooked is engineering the delivered cargo sequence itself. For example, cargos might contain sequence motifs like cryptic splice sites, which can severely inhibit delivery by disrupting the viral genome through splicing. One avenue that has been successfully demonstrated to overcome this challenge is the intentional inclusion of several strong intronic sequences (Gleba et al., 2004). This approach is thought to ensure the intact viral genome is the predominant cytoplasmic product from nuclear expression of the viral genome from a T-DNA. Additionally, the sequences being delivered via viral vectors are rarely completely viral in nature. As a result, the sequence characteristics such as GC content and secondary structure tend to be significantly different from viral sequences (Ben-Shaul and Gelbart, 2015). The incorporation of foreign sequences might disrupt the native secondary and tertiary structure of the virus and it has been shown that this structure is very important for effective expression and encapsidation of the viral genome (Chen et al., 2010; Cho and Kim, 2012). As encapisdation plays a role in viral genome stability and effective systemic movement for some viruses (Lee et al., 2011), designing novel strategies to reengineer cargo sequences to make them more “viral-like” could be another way to improve expression from viral vectors. The efficacy of increasing the GC content of cargos to stabilize them in viral vectors was recently demonstrated (Torti et al., 2021). The success of refactoring a cargo sequences by including strong introns and higher GC content indicates there might be more promising strategies to improve cargo stability and expression through sequence modification.

## Engineering Improved RNA Silencing Suppression

One of the central challenges to the initial establishment of an infection and maintenance of a consistent viral titer over time is RNA silencing by the host plant. This phenomenon is triggered by the recognition of double stranded viral RNA, which occurs either during replication or before encapsidation due to the high degree of secondary structure in the viral genome (Carrington et al., 2001). RNA viruses that are well adapted to their hosts encode proteins that suppress RNA silencing. One approach used to allow the successful establishment of infection by a virus in a non-native host is to transiently express RNA silencing suppressors at the time of infection (Li and Wang, 2018). This improves the chances of infection but does not affect long term silencing by the plant. A more permanent solution is to knock out genes involved in the RNA silencing pathway. It was demonstrated that knocking out the RdRP1 protein in *A. thaliana* led to higher viral titers and more persistent infections with tobamoviruses and tobraviruses (Yu et al., 2003). While this approach does improve infection and persistence of viral vectors, it makes the host vulnerable to infection by any viruses in the environment and is therefore a sub-optimal solution outside of controlled settings. It also might create confounding effects that complicate interpretation of results observed from the synthetic biology interventions being delivered, as these pathways tend to be involved in more than just viral defense.

## Engineering Effective Containment Mechanisms

The use of agents capable of self-replication, such as RNA viruses, necessitates a careful consideration of effective biocontainment mechanisms, as well as strategies for virus clearing in case of escape. In laboratory settings, these vectors are used in isolated chambers free of insects or other vectors to prevent potential environmental escapes. For certain viruses, additional layers of containment have been incorporated, such as mutations in the coat proteins to prevent transmission (Touriño et al., 2008). However, these kinds of interventions sometimes come at the cost of the efficacy of viral movement (Valentine et al., 2004). Another potential control mechanism, which has shown promise in mammalian viral vectors but has yet to be tested in plant viruses, is the incorporation of aptazymes into the viral genome. Aptazymes are RNA motifs composed of a small molecule binding aptamer domain and a catalytic ribozyme domain (Zhong et al., 2016). Aptazymes adopt a secondary structure which catalyzes cleavage of the RNA backbone in the presence of a specific trigger molecule. When incorporated into the viral genome these motifs can be triggered to clear the virus through cleavage of the linear genome. Aptamers that respond to several agrochemicals, which are optimized for penetration into plant tissues, have been identified (Liu et al., 2019). These could be used to create aptazymes that can be triggered effectively through chemical treatment to clear viral vectors in a plant.

However, thanks to the low fidelity and rapid replication of RNA viral vectors and the strong selective pressure to mutate the control mechanism, there is a chance of escape from these strategies over time. Thus, it is important to engineer these kinds of control mechanisms to be robust to mutation, for example by layering multiple independent containment mechanisms so that all would have to fail simultaneously to allow escape. Beyond incorporating containment mechanisms, another avenue to engineer effective biocontainment is to fundamentally reengineer the virus and relocate the viral replication machinery into the plant genome (Gleba et al., 2004; Bedoya et al., 2010). Viral vectors deficient in viral replicase could then be used for gene delivery, removing the capacity for replication outside the target host. While this approach does require creation of an initial plant line, it would provide a strong layer of containment. Thoroughly testing the efficacy and stability of these various containment mechanisms, both separately and in conjunction with one another, is essential in translating the transformative power of RNA vector-based plant synthetic biology from the lab to the field.

## Broadening Access to Plant Synthetic Biology With RNA Vectors

Science is founded on the principle that acquired knowledge should be available for all. In practice, however, science is filled with the same inequities that exist across the world. This is, in part, driven by the kinds of enabling tools we develop to move science forward. The focus on expensive and technically challenging methods to deploy plant synthetic biology, such as plant transformation, have restricted its use outside of easily transformed model plants like *A. thaliana* to resource rich settings. By limiting who is doing this kind of science, we also limit the kinds of questions these tools are used to ask, leaving science poorer for it. RNA vectors, once sufficiently developed, have the potential to dramatically lower both the material cost and person hours required to implement synthetic biology-based strategies to study plants. When coupled with lower cost reporters (Khakhar et al., 2020), they have the potential to usher in a new era of inclusivity in plant synthetic biology and bring a diversity of voices to this emerging community.

## Conclusion

Using RNA viral vectors to deploy plant synthetic biology tools can collapse experimental timelines, especially in crop plants. RNA viral vectors can also be easily and cheaply scaled, allowing the interrogation of biology at a scale that was previously unfeasible. For example, they could be used to rapidly tune the expression of every gene in a metabolic pathway and identify expression ratios that optimize metabolic flux to desirable products like vitamins or pharmaceuticals. This approach could also be applied to rapidly interrogate how changes in the expression level or methylation state of developmental regulators translate to whole plant phenotypes. The broad host range of viral vectors means they could expand the use of plant synthetic biology beyond model species. All of these potential benefits highlight the need to invest in the development of these technologies, both through the exploration of some of the engineering strategies we suggest here, as well as through deeper study of plant virome. Combining an expanded understanding of the mechanistic basis of plant virology with the engineering principles of synthetic biology will enable the construction of gene delivery tools that might one day obviate the need for transgenesis. While this next generation of tools is being developed, existing RNA viral vectors will enable the rapid generation of hypotheses about the mechanistic underpinnings of biological phenomena, which can be further interrogated with stable transgenic lines. In this way RNA vectors will accelerate the pace of biological discovery in plants and, once demonstrated to be safe and containable, allow the rapid translation of these insights into crops in the field.

## Data Availability Statement

The original contributions presented in the study are included in the article/supplementary material, further inquiries can be directed to the corresponding author.

## Author Contributions

AK wrote the manuscript and prepared the figures. DV provided the editorial input. Both authors contributed to the article and approved the submitted version.

## Conflict of Interest

The authors declare that the research was conducted in the absence of any commercial or financial relationships that could be construed as a potential conflict of interest.
